# *Pueraria tuberosa*: A Review on Traditional Uses, Pharmacology, and Phytochemistry

**DOI:** 10.3389/fphar.2020.582506

**Published:** 2021-01-27

**Authors:** Ram Bharti, Bhupinder Singh Chopra, Sachin Raut, Neeraj Khatri

**Affiliations:** ^1^ IMTECH Centre for Animal Resources & Experimentation (iCARE), Council of Scientific and Industrial Research-Institute of Microbial Technology (CSIR-IMTECH), Chandigarh, India; ^2^ Academy of Scientific and Innovative Research (AcSIR), Ghaziabad, India

**Keywords:** *in vivo* studies, pharmacological properties, phytochemical constituents, traditional uses, *Pueraria tuberosa*

## Abstract

*Pueraria* *tuberosa* (Roxb. ex Willd.) DC. (Fabaceae), also known as Indian Kudzu (vidari kand), is a perennial herb distributed throughout India and other Asian countries. Traditionally, tuber and leaves of this plant have extensively been reported for nutritional and medicinal properties in Ayurveda as well as in Chinese traditional practices. The objective of the present review is to compile and update the published data on traditional uses, pharmacological potential, and phytochemistry of compounds isolated from the plant *Pueraria tuberosa*. *P. tuberosa* extracts and its purified compounds possess multiple activities such as anticancer, anticonvulsant, antidiabetic, antifertility, anti-inflammatory, antioxidant, anti-stress, antiulcerogenic, cardioprotective, hypolipidemic, hepatoprotective, immunomodulatory, nephroprotective, nootropic, neuroprotective, and wound healing. Tuber and leaf extracts of *P. tuberosa* contain several bioactive constituents such as puerarin, daidzein, genistein, quercetin, irisolidone, biochanin A, biochanin B, isoorientin, and mangiferin, which possess an extensive range of pharmacological activities. The extensive range of pharmacological properties of *P. tuberosa* provides opportunities for further investigation and presents a new approach for the treatment of ailments. Many phytochemicals have been identified and characterized from *P. tuberosa*; however, some of them are still unexplored, and there is no supporting data for their activities and exact mechanisms of action. Therefore, further investigations are warranted to unravel the mechanisms of action of individual constituents of this plant.

## Introduction

**FIGURE 1:**
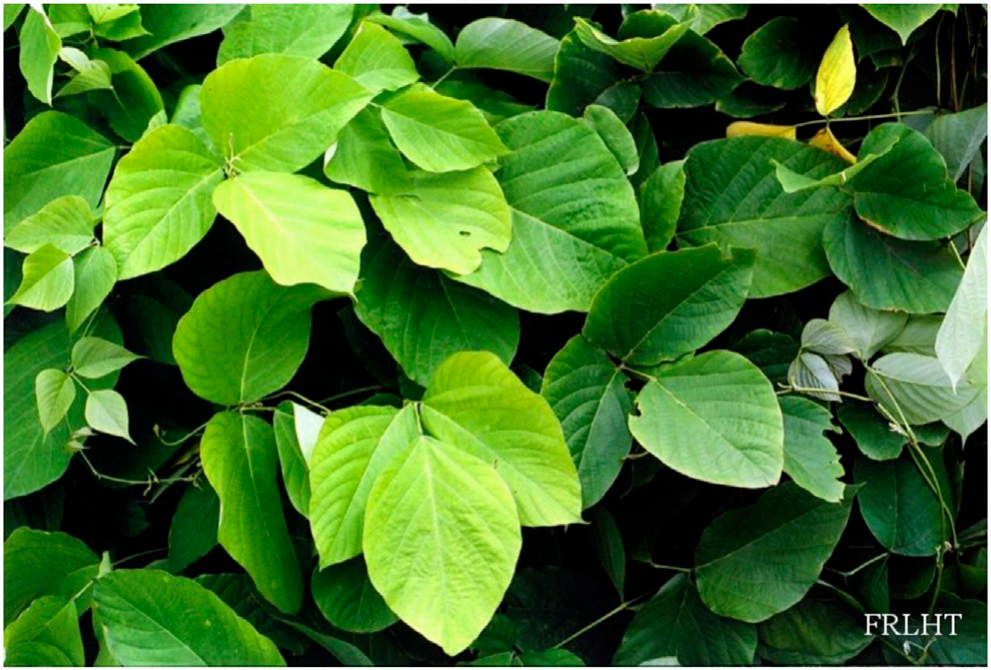
*Pueraria tuberosa* (Roxb. ex Willd.) DC. (Fabaceae): (1) Leaf. (2–4) Tuber.

**FIGURE 2:**
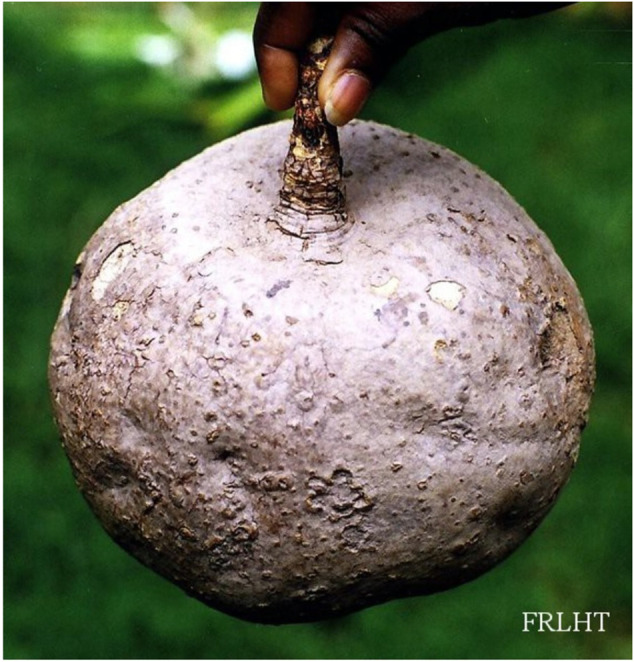
Indian Medicinal Plant database.

**FIGURE 3:**
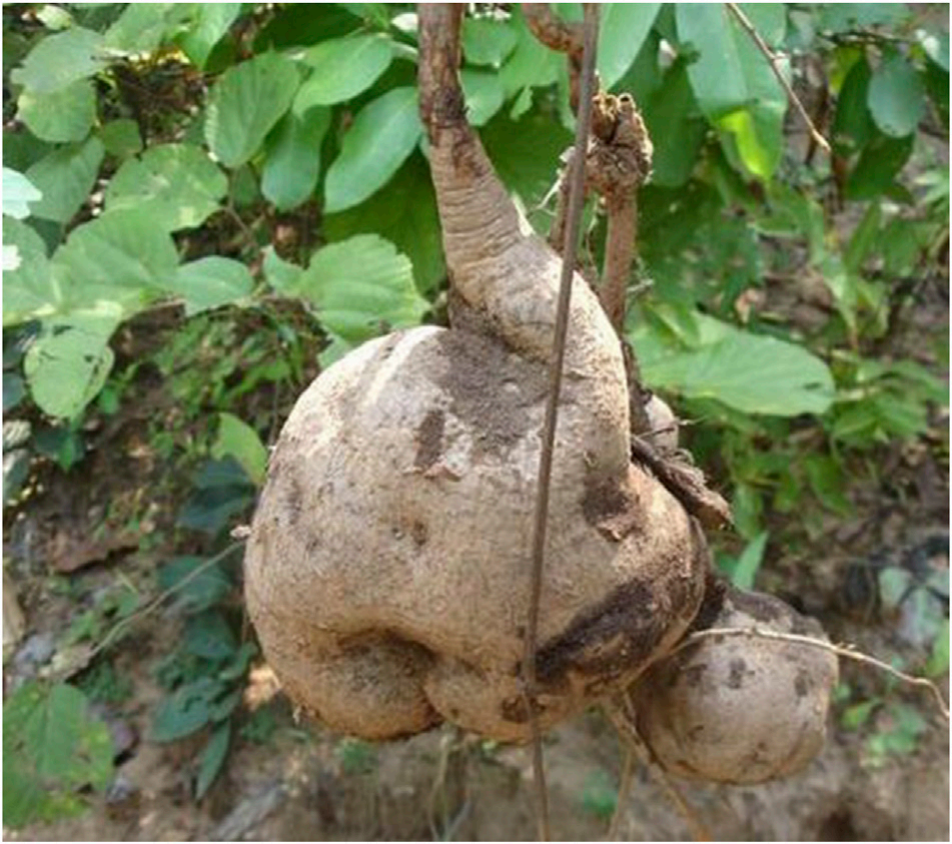
Pankaj Oudhia/https://www.discoverlife.org.

**FIGURE 4:**
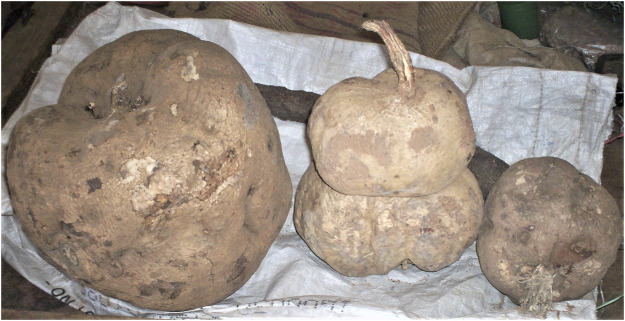
“Pueraria tuberosa (Willd.) DC. Vidari Kand, Patal Kumrha”, by Ravi Upadhyay, https://indiabiodiversity.org/observation/show/269544, licensed under CC BY 3.0).

As per the World Health Organization (WHO) estimation, about 65–80% of people all over the world seek herbal therapies to cure primary health conditions (Robinson and Zhang, 2011). Surprisingly, only 15% of the global flora has been assessed for pharmacological potential (De Luca et al., 2012). WHO has published four volumes of the monographs on selected medicinal plants to support the research in the field of herbal medicine (WHO, 2009). In India, Ayurveda, Unani, Siddha, Homeopathy, and Folk medicine are commonly used as traditional alternative medicine practices for treating different ailments. Among the modern civilizations, India has long been known for its rich treasure of medicinal plants, and about more than 7,000 plant remedies have been categorized and documented by the AYUSH system of medicine (National Medicinal Plants Board, Government of India, 2020). One of the medicinally important plants discussed in this review is *Pueraria tuberosa* (Roxb. ex Willd.) DC. (Fabaceae), also known as Indian Kudzu (vidari kand). It is a rapidly growing large perennial climber with big tuberous roots (Figures 1–4) (Indian Medicinal Plant Database) and is distributed throughout India, Pakistan, and Nepal (Keung, 2002). Lianas of *P. tuberosa* has also been found to grow at 4,000 feet in the Himalayan mountain series (Pueraria tuberosa—Vikaspedia, 2020). In Ayurveda, it is known as vidari (vidari kand). The tuber of this plant is sweet (Ayurvedic pharmacopoeia of India, 2001) and is widely used in the treatment of fever, menorrhagia, skin diseases, wounds, bronchial asthma, and jaundice. Apart from the traditional uses of this plant as mentioned in ancient literature like Sushruta Samhita (Sanskrit: सुश्रुत संहिता), several studies have been reported on different pharmacological activities of *P. tuberosa* extracts and its purified compounds, viz., anticancer (Adedapo et al., 2017), anticonvulsant (Basavaraj et al., 2011), antidiabetic (Oza and Kulkarni, 2018a), antifertility (Gupta et al., 2005), anti-inflammatory (Tripathi et al., 2013), antioxidant (Shukla et al., 2018a), anti-stress (Verma et al., 2012), antiulcerogenic (Gindi et al., 2010), cardioprotective (Patel et al., 2018), hypolipidemic (Tanwar et al., 2008), hepatoprotective (Xia et al., 2013), immunomodulatory (Patel et al., 2016), nephroprotective (Shukla et al., 2018b), nootropic (Rao et al., 2008), neuroprotective (Xing et al., 2011), and wound healing activities (Kambhoja and Murthy, 2007). Previously, Maji et al. (2014) broadly highlighted the phytochemical and therapeutic potential of *P. tuberosa* in various pharmacological activities. However, the information about the doses of plant extracts used and the models implied for the studies (*in vitro* or *in vivo*) in different pharmacological activities was missing. In addition, chemical structures of only few phytoconstituents isolated from *P. tuberosa* have been given. Therefore, this review is aimed to provide an up-to-date summary of the literature on traditional uses, doses, and types of studies used to confirm pharmacological activities and phytochemical constituents isolated from *P. tuberosa* plant with their chemical structures and IUPAC names.

## Methodology

Relevant literature for this review on *P. tuberosa* has been sourced from PubMed, ScienceDirect, Web of Science, PubChem, Google Scholar, SciFinder, and Scopus database. The articles published in English before September 2020 on traditional uses, pharmacology of extracts, and various phytoconstituents isolated from different parts of *P. tuberosa* were included in this review. The keywords used for retrieving relevant studies were *Pueraria tuberosa* plant, Indian Kudzu, vidari kand, tuber extract, traditional uses, phytochemical constituents, pharmacological activity, and *in silico*, *in vitro*, and *in vivo* studies.

Data inclusion criteria included (a) published/peer-reviewed scientific manuscripts; (b) ethnopharmacological studies; (c) tuber extracts with different solvents; (d) studies on the mechanism of actions of plant extracts and their phytoconstituents; (e) *in silico*, *in vitro*, and *in vivo* studies. Exclusion criteria included (a) repetitive studies and information not meeting the inclusion criteria; (b) studies performed with extracts of other *Pueraria* species; (c) opinion to the editors, case studies, abstracts of the conferences, any unpublished data, and reports.

### Synonyms (Ayurvedic pharmacopoeia of India, 2001)

Assamese: Bhedeleton, Bhuikumra
 Bengali: Bhuinkumra, Bhumikusmanda, Vidari
 English: Indian kudzu
 Gujrati: Bhoikolu, Bhonykoru, Eagio, Sakharvel, Vidarikanta,
 Hindi: विदारीकंद (Vidarikanda), बनकुमड़ा (Bankumara)
 Kannada: Gumadi belli, Gumadigida, Nelagumbala Gudde, Nelagumbala, Nelagumbula
 Malayalam: Mudakku
 Marathi: Bhuikohala, Ghodvel
 Oriya: Bhuiankakharu
 Punjabi: Siali, Surala
 Sanskrit: भूमिकुष्माण्ड (Bhumikusmanda), गजवाजिप्रिया (Gajavajipriya), कन्दपलाश (Kandapalash), स्वादुकन्दा (Svadukanda), विदारी (Vidari), इक्षुगन्धा (Iksu-Gandha).
 Tamil: Nilapoosani
 Telugu: Darigummadi, Nelagummuda

### Scientific Classification (Rawtal et al., 2019)

 Kingdom: Plantae
 Subkingdom: Trachebionta
 Superdivision: Spermatophyta
 Division: Magnoliophyta
 Subclass: Rosidae
 Order: Fabales
 Family: Fabaceae
 Genus: *Pueraria* DC.
 Species: *Pueraria tuberosa*

### Traditional Uses

In Ayurveda, vidari kand (*Pueraria tuberosa*) has been described as a plant having good nutritional value. Besides, the plant also possesses aphrodisiac, diuretic, galactagogue (Kirtikar and Basu, 1935), energizing (Maji et al., 2014), and spermatogenic (Chauhan et al., 2013) properties. It has been prescribed for treatment for all three doshas (i.e., for the complications of three different energies, viz., Vata, Kapha, and Pitta) of human body (Ayurvedic pharmacopoeia of India, 1999; Dalal et al., 2013). The powdered form of tuber is primarily used in combination with cow’s milk as a galactagogue agent to abrogate lack of milk production after childbirth and also as an anabolic agent along with *Piper longum* L. (Piperaceae) powder to cure malnutrition in children. For relieving excessive menstruation, the powder is used with honey. A mixture of powdered *P. tuberosa* and wheat or barley fried in ghee (clarified butter) with milk has been advised for sexual enervation and strength. For spermatorrhoea, fresh tuber juice of this plant with cumin seeds and sugar has been used therapeutically (Puri, 2003).

Traditionally, *P. tuberosa* has been used along with other medicinal plants in different combinations to prepare therapeutic Ayurvedic formulation. Some of the important Ayurvedic formulations utilizing *P. tuberosa* are “Ashwagandharishta”, a traditional remedy for epilepsy (Tanna et al., 2012), “Maha visagarbha taila”, a traditional remedy for sciatica and joint disorders (Kumawat et al., 2017), and “Nityananda rasa”, “Sarasvatarista”, “Satavaryadi ghrta” (Ayurvedic pharmacopoeia of India, 2001), “Marma gutika” (Kumar, 2016), and “Vidaryadi ghrita” (Sharma et al., 2018).

Traditional uses of *Pueraria* species, namely, *Pueraria montana* var. *thomsonii* (Benth.) (Fabaceae) and *Pueraria montana* var. *lobata* (Willd.) (Fabaceae), have been reported for their medicinal properties such as antiemetic, antitoxic, cold, countering the effect of alcohol abuse, anti-stress agent, neck stiffness, hypohidrosis, migraines, hypoglycemia, and certain cardiovascular diseases in the Chinese Medicinal Herbs, a book written by Li Shih Chen (Li, 2003; Croom, 2004).

### Pharmacology

**FIGURE 5:**
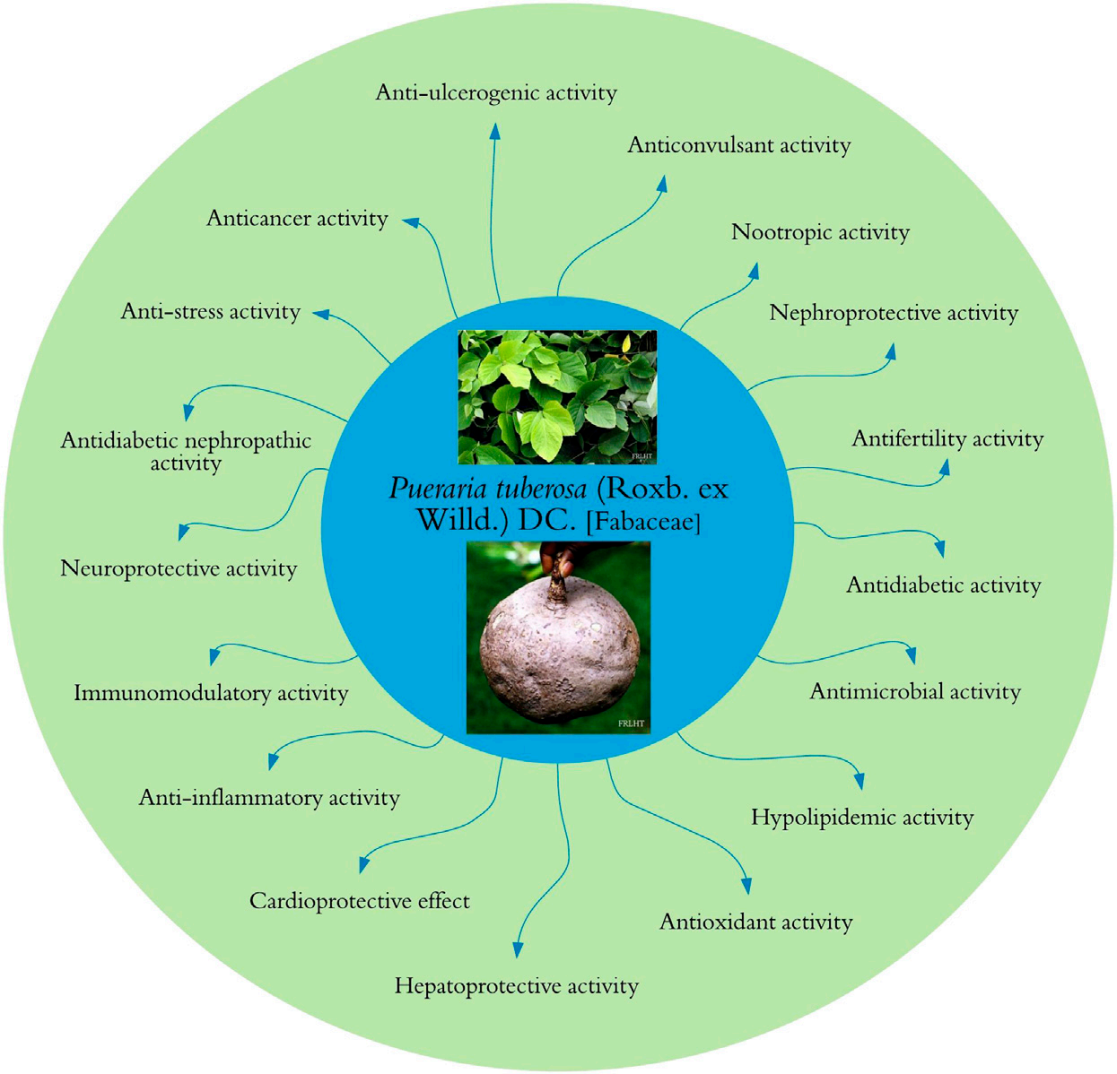
Pharmacological activities of *Pueraria tuberosa*.

In phytopharmacological/ethnopharmacological research, scientific community should follow best practices in designing and conducting studies and reporting the results of analyzing pharmacological properties of the plant extracts and compounds of natural origin (Heinrich et al., 2020). Therefore, while reporting biological activities of any plant/herbal product, detailed information about the characterization of the plant extracts, their phytoconstituents, doses, duration of treatment, type of models used in the studies, toxicological data, and so forth should be clearly presented for the benefit of research community (Heinrich et al., 2020). Various pharmacological activities of the tuber extracts of *P. tuberosa* have been explored, and a graphical summary of these activities is shown in Figure 5 and Table 1.

**TABLE 1:** Pharmacological activities of tuber extract of *Pueraria tuberosa*.

Extract	Dose tested	Pharmacological activity	Model used for study (*in vivo* or *in vitro*)	Reference
Aqueous	50 mg/100 g b/w	Antidiabetic	*In vivo*	Srivastava et al. (2015); Srivastava et al. (2017); Srivastava et al. (2018); Srivastava et al. (2019)
	50 mg/100 g b/w for 35 days			
	50 mg/100 g b/w for 10 days			
Ethanol	100–400 mg/kg b/w for 5 days	Immunomodulatory	*In vivo*	Patel et al. (2016)
Tuber powder	250 mg/kg b/w	Immunomodulatory	*In vivo*	Shilpashree et al. (2015)
Aqueous	250 mg/ml given orally to rats for 14 days	Hepatoprotective	*In vivo*	Pandey et al. (2019)
Ethanol and methanol	125, 250, 500, and 1,000 μg/ml	Antioxidant	*In vitro*	Likhitkar and Pande (2017)
Aqueous	200, 400, and 700 μg/ml for 24, 48, and 72 h	Anticancer	*In vitro*	Adedapo et al. (2017)
Hydroalcoholic	64 and 128 µg/ml for 24 h	Anticancer	*In vitro*	Aruna et al. (2018)
Ethyl acetate	31.5–500 μg/ml for 72 h	Anticancer	*In vitro*	Satpathy et al. (2020)
Aqueous	50–100 mg/100 g b/w for 20 days	Antidiabetic nephropathy	*In vivo*	Shukla et al. (2017); (2018a); (2018b)
Hydroalcoholic	20–40 mg/100 g b/w for 14 days	Antidiabetic nephropathy	*In vivo*	Tripathi et al. (2017)
Methanolic	20 mg/kg b/w for 14 days and 40 mg/kg b/w for 7 days	Antidiabetic nephropathy	*In vivo*	Yadav et al. (2019)
Methanolic	20 and 40 mg/100 g b/w for 2 days	Nephroprotective	*In vivo*	Yadav et al. (2016a)
Butanol and ethyl acetate	50 mg/100 g b/w for 5 days	Nephroprotective	*In vivo*	Yadav et al. (2016b)
Methanolic	200 mg/ml	Antibacterial	*In vitro*	Pandya et al. (2019)
Hydroalcoholic	50, 100, and 200 mg/kg b/w for 30 days	Neuroprotective	*In vivo*	Umarani et al. (2016)

b/w: body weight.

### Nephroprotective Activity

Several studies have shown that *P. tuberosa* plant possesses nephroprotective activities. Oral administration of methanolic tuber extract to cisplatin- (8 mg/kg body weight) induced kidney damaged rats showed a dose-dependent protective effect (Nagwani and Tripathi, 2010). Tuber extract significantly reduced blood urea nitrogen, serum creatinine, glutathione, and superoxide dismutase (SOD) levels. The extract could control deoxyribonucleic acid (DNA) damage and catalase activities, cellular necrosis, and tubular swelling and prevent coagulation of proteins, in contrast to the control group. The nephroprotection of tuber extract of the plant has been attributed to its free radical scavenging activity (Nagwani and Tripathi, 2010). Feeding of biscuits made up of powder of *P. tuberosa* tuber for 10 days showed significant recovery in cisplatin-induced nephrotoxicity in Swiss mice. However, at higher dose, aspartate aminotransferase and alanine aminotransferase levels were temporarily elevated, so monitoring of liver functions, periodically, is imperative when continuing this regimen for longer periods such as a food supplement for cancer patients undertaking cisplatin chemotherapy (Tripathi et al., 2012). The methanolic extract of *P. tuberosa* ameliorated glycerol-induced acute kidney injury in rats by affecting the lipid peroxidation, SOD, and catalase activity with a lesser accumulation of hyaline casts and a lesser degree of tubular necrosis on histology of the kidney (Yadav et al., 2016a). Water decoction of *P. tuberosa* has also been reported to significantly reverse cisplatin-induced nephrotoxicity in rats (Yadav et al., 2016b). Hydroalcoholic tuber extracts of *P. tuberosa* showed nephroprotective activity in sodium arsenate- (1 mg/kg body weight) induced oxidative kidney tissue damage in rats (Rani et al., 2017). The nephroprotective effect through free radical scavenging activity was supported in a study, where streptozotocin- (STZ-) induced diabetic nephropathic rats, treated with aqueous tuber extract of *P. tuberosa*, exhibited an upsurge in activity of antioxidant enzymes, lowered oxidative stress, apoptosis, and urinary albumin excretion in a concentration-dependent manner (Shukla et al., 2018a). Methanolic tuber extract of the plant showed substantial protection in diabetic nephropathy induced by the administration of alloxan in rats (120 mg/kg body weight) by decreasing urea and creatinine and improving physiology of the kidney (Yadav et al., 2019). The supplementation of tuber extract of the *P. tuberosa* showed protection of kidney from oxidative stress and cellular injury. It also improved kidney physiology and parameters of kidney function test by reducing cellular apoptosis. These studies indicate that *P. tuberosa* extracts have nephron-protective potential and might lead to promising therapeutic agents for treating kidney diseases.

#### Antioxidant Activity

Methanolic and hexane tuber extract of *P. tuberosa* exhibited a strong free radical scavenging activity in a concentration-dependent fashion. These results showed that the methanolic extract of this plant exhibited better activity than the hexane extract in trapping hydroxyl radicals and inhibited lipid peroxidation, which indicated potent antioxidant property (Pandey et al., 2007). Hot water tuber extract of the plant *P. tuberosa*, supplemented with milk in Swiss mice, showed potent antioxidant activities in liver and red blood cells. Besides, a remarkable difference in glutathione levels was also observed in the control (172 μg/ml) and supplemented groups (*P. tuberosa*: 1,212 μg/ml and *P. tuberosa* + milk: 1,308.2 μg/ml). *P. tuberosa* along with milk has antioxidant property as evidenced by higher phagocytic activity, increased immunoglobulin levels, and reduced glutathione and lipid peroxidation (Sawale et al., 2013). *P. tuberosa* extracted with chloroform, acetone, methanol, and hot water was used to determine its antioxidant potential by using ferric reducing antioxidant power (FRAP) assay, metal chelating, phosphomolybdenum, and free radical scavenging using DPPH (2,2′-diphenyl-1-picrylhydrazyl radical) and ABTS (3-ethylbenzothiazoline-6-sulfonic acid) assay. The results showed that acetone extract of *P. tuberosa* has potent antioxidant activity (Viji and Paulsamy, 2015).

#### Antidiabetic Activity

Oral gavage of ethyl acetate tuber extract of *P. tuberosa* (250 mg/kg body weight) to alloxan-induced diabetic rats for seven days showed a pronounced decrease in blood glucose levels (Raghuwanshi and Jain, 2011). Studies suggested that chloroform, petroleum ether, ethanol, and aqueous tuber extracts of *P. tuberosa* confer significant antidiabetic activity in STZ- (50 mg/kg body weight) induced diabetic rats by a single intraperitoneal injection (Tripathi and Kohli, 2013). Water extract of root of *P. tuberosa* showed significant inhibition of dipeptidyl peptidase-4 (DPP-IV) that causes an enhanced half-life of active glucagon-like peptide-1 hormone. This hormone regulates glucose-dependent insulin release from β-cells of the pancreas in rats (Srivastava et al., 2015). In Srivastava et al.’s next study, they found that *P. tuberosa* water extract increased the glucose homeostatic potential through DPP-IV inhibitory pathway and the bioactive components robinin and puerarone, and this inhibitory activity was also confirmed by *in silico* molecular docking (Srivastava et al., 2017). Aqueous extract of tuber of *P. tuberosa* has further been reported to act as incretin receptor agonist and downregulated β-cells apoptosis and protected STZ-induced diabetes in rats (Srivastava et al., 2018). Aqueous tuber extract of the plant showed an elevated expression of nephrin and SOD and a declined expression of cysteinyl aspartate specific proteinase 3 (caspase-3), interleukin 6 (IL-6), nuclear factor kappa B (NF-κB), protein kinase C epsilon type (PKCε), tumor necrosis factor alpha (TNF-α), vascular endothelial growth factor (VEGF), matrix metalloproteinase-9 (MMP-9), and hypoxia-inducible factor 1-alpha in STZ-induced diabetic rats (Srivastava et al., 2019). In another experiment, it has been shown that administration of *P. tuberosa* water extract in alloxan-induced rat diabetic model resulted in decrease in SGOT (serum glutamic oxaloacetic transaminase), SGPT (serum glutamic pyruvic transaminase), and alkaline phosphates level and improved deformed hepatocytes and significant decrease in blood glucose levels as well as apoptosis (Pandey et al., 2019). The tuber extract contains different bioactive compounds that may act as agonists on glucagon-like peptide-1 hormone released from intestine and can also protect β-cells of the pancreas. It also resulted in decreased expression of different inflammatory and apoptotic markers during hypoxic injury to β-cells as evidenced by decreased apoptosis of β-cells. The extract also inhibited DPP-IV enzyme as an incretins receptor agonist, and hence it is emanating from the above studies that *P. tuberosa* has antidiabetic potential.

#### Anti-Stress Activity

Adult male Wistar rats subjected to cold immobilization stress, pretreated with 70% hydroethanolic tuber extract of *P. tuberosa* (200 and 400 mg/kg body weight) for 5 days, showed significant protection from gastric mucosal damage, reduced corticosterone level in the blood, and no enlargement of spleen and adrenals as compared to *Withania somnifera* (L.) Dunal (Solanaceae) rhizome extract (100 mg/kg body weight). These studies established the anti-stress effect of *P. tuberosa* (Pramanik et al., 2011). In a human trial, hypertensive patients were divided into two groups: group 1 was given capsules with 0.75 g tuber powder, whereas group 2 was given placebo capsules with lactose powder administered for 12 weeks. Group 1, treated with 1.5 g (twice a day) tuber powder of *P. tuberosa* for 12 weeks, showed a gradual decrease in systolic, diastolic, and mean blood pressure as well as a tolerant decrease in fibrinogen and increased plasma fibrinolytic activity (Verma et al., 2012). In stress-mediated disorders, the hypothalamic-pituitary-adrenal (HPA) axis is dysregulated which changes the levels of corticosteroids in plasma and monoamine in the brain. The extract of this plant might act on mucosal layer of the gastrointestinal, cardiovascular, and nervous (HPA) system, suggestive of anti-stress activity by a reduction in stress hormones.

#### Antidiabetic Nephropathic Activity

STZ-induced diabetic rats with nephropathy were given tuber extract of *P. tuberosa* (30 mg/100 g, body weight) for 20 days and exhibited a significant reduced severity of diabetic nephropathy by enhanced expression and activity of MMP-9 and degrading the accumulation of extracellular matrix in kidney tissue (Tripathi et al., 2017). Levels of nephrin, a biomarker of early glomerular injury, in the kidney of diabetic nephropathic rats were restored after treatment with tuber extract of *P. tuberosa* (Shukla et al., 2017). The diabetic nephropathic inflammatory response is mediated by NF-κB and its activated phosphorylated derivative (pNF-κB). Improved levels of these transcription factors and inflammatory cytokines (IL-6 and TNF-α) in the kidney of STZ-induced (55 mg/kg body weight) diabetic nephropathic rats were observed, and treatment with extracts from the tuber of *P. tuberosa* significantly negated these changes in a dose-dependent manner (Shukla et al., 2018b). Amelioration of renal damage was evaluated by renal functional tests, histopathology, and oxidative stress in alloxan-induced diabetic nephropathy. *P. tuberosa* methanolic extract showed renal protection by decreasing urea and creatinine and improved kidney physiology and histopathology changes through antioxidant mechanisms (Yadav et al., 2019). These studies are indicative of nephro-protection offered by *P. tuberosa* in diabetic nephropathy; however, this protective effect needs to be further explored, including studies on the protection of renal and glomerular cells mediated by different signaling pathway in the antidiabetic nephropathy.

#### Anti-Inflammatory Activity

The ethyl acetate and methanolic tuber extracts of *P. tuberosa* showed considerable anti-inflammatory potential compared to the control and standard drugs, ibuprofen, and nitrofurazone ointment in the rat paw edema method (Kambhoja and Murthy, 2007). The methanolic tuber extract of the plant significantly prevented the carrageenan-induced inflammation by lowering the glutathione content, catalase, SOD activity, and enhancing lipid peroxidase and C-reactive proteins in rats in a sequential manner (Tripathi et al., 2013). Isoorientin, isolated from the tuber of *P. tuberosa* plant, showed significant anti-inflammatory activity in LPS-treated mouse macrophage (RAW 264.7) cell line. It was also effective against carrageenan-induced inflammation on paw edema and air pouch mouse models. These studies revealed the downregulation in the expression of proinflammatory genes such as inducible nitric oxide synthase (iNOS), cyclooxygenase-2 (COX-2), TNF-α, and inactivation of NF-κB. Moreover, there was activation of antioxidant enzymes, catalase and glutathione-S-transferase (Anilkumar et al., 2017). The anti-inflammatory property of extracts of *P. tuberosa* in these studies appears to be mediated by lipid peroxidation, inactivation of the NF-κB pathway, and downregulation of proinflammatory cytokines.

#### Immunomodulatory Activity

Immunomodulatory activities of plant extract (0.4%) with milk as a carrier given to Swiss mice for 28 days were evaluated. The result showed a significantly higher phagocytic activity and immunoglobulin concentration, reduced glutathione content, and thiobarbituric acid reactive substances level compared to the control (Sawale et al., 2013). Reversed phase high-performance liquid chromatography (RP-HPLC) analysis of ethanolic tuber extract of the plant revealed that bioactive compounds involved in the immunomodulatory activities are genistein (1.37%), daidzein (1.70%), and puerarin (8.31%). Oral administration of these extracts builds up innate and humoral immune responses against sheep red blood cells challenged rats (Maji et al., 2014). The immunomodulatory activity of petroleum ether extract of *P. tuberosa* was evaluated by carbon clearance assay (Granulopectic index). The extract and *Withania somnifera* (L.) Dunal (Solanaceae) at 250 mg/kg body weight (Medicinal Plant Names Services, e) exhibited enhanced phagocytic activity of peritoneal macrophages to clear the carbon particles (Shilpashree et al., 2015). The ethanolic extract of tuber increased the phagocytic activity of macrophages in the mice model. The extract also inhibited both the cell mediated and humoral immunity, which supports its potent immunomodulatory activity (Patel et al., 2016).

#### Anticancer Activity

There is no significant toxicity of mangiferin isolated from tuber of *P. tuberosa* on normal cell lines (mouse fibroblast NIH-3T3, RAW 264.7, HEK293, and mouse lymphocytes) in cell viability assay *in vitro*; however, it is cytotoxic to various cancer cell lines like K562, MCF7, HEPG2, Jurkat cells, and A549 (Bulugonda et al., 2017). Furthermore, the anticancer and apoptotic potential of the hydroalcoholic tuber extract of *P. tuberosa* was investigated by cell viability assay. The extract showed a 50% inhibition of cell viability against human colon carcinoma (HT-29) cells at a concentration of 63.91 µg/ml. Cells also exhibited DNA fragmentation that is the hallmark of apoptosis, apoptotic cell death, and increased expression of certain proapoptotic genes (Aruna et al., 2018). The silver nanoparticles biosynthesized with aqueous extract of the *P. tuberosa* showed *in vitro* anticancer potential on different cancer cell lines (breast MCF-7 and MDA-MB-231; ovarian SKOV-3; brain U-87 cancer). However, the mechanism behind this activity needs exploration for therapeutic use (Satpathy et al., 2018). Antioxidant-enriched fraction also exhibited *in vitro* cytotoxicity in the breast (MCF-7 and MDA-MB-231) and ovarian (SKOV-3) cancer cells (Satpathy et al., 2020).

### Other Pharmacological Properties

*P. tuberosa* has been attributed as one of the most sought plants that proved to be effective against multiple diseases and ailments. Alcoholic and aqueous extracts of *P. tuberosa* tuber were studied for nootropic effect in mice and rat models of amnesia induced by scopolamine and diazepam. The inflexion ratio observed was considerably high and comparable with piracetam, the standard drug in an elevated plus-maze experiment. Flavonoids present in the *P. tuberosa* tuber extracts have been reported for nootropic effect by interacting with cholinergic, adrenergic, serotonergic, and GABAnergic system (Rao et al., 2008). The neuroprotective properties of this plant were also studied in chronic foot-shock stressed rat model showing unpredictable and inescapable nature of physiological malfunctions, increase in anxiety level, decrease in male sexual indices, and behavioral changes. All these symptoms were abolished by this plant’s tuber extract (Pramanik et al., 2010). Neurotoxicity induced by sodium arsenate was ameliorated by hydroalcoholic extract which strengthens its memory and restores muscle strength and locomotor activity. Biochemical and histopathological changes are suggestive of the protective property of the extract in maintaining normal functional status of the brain in arsenate neurotoxicity (Umarani et al., 2016).

Alcoholic tuber extract of *P. tuberosa* was studied for anticonvulsant activity in pentalene tetrazole, strychnine, and maximal electroshock-induced convulsions in animals. Different doses of the extract (50, 100, and 200 mg/kg body weight) were compared with the standard drug, diazepam (5 mg/kg body weight). The medium and high doses exhibited potent anticonvulsant activity as compared to the control group (Basavaraj et al., 2011). The ethanolic and methanolic extract of leaf, stem, and tuber of *P. tuberosa* showed a wide range of antimicrobial activity against bacteria, *Escherichia coli*, *Bacillus cereus*, *Salmonella paratyphi*, and *Staphylococcus aureus*, as well as fungus, *Candida albicans*, *Aspergillus fumigates*, and *Alternaria solani*, on agar diffusion assay (Sadguna et al., 2015). The tuber extracts of *P. tuberosa* with different solvents exhibited a wide range of antimicrobial activity on selected bacterial and fungal pathogens (Aruna et al., 2016). The chloroform and water extracts of tuber of *P. tuberosa* showed significant antibacterial activity against *Klebsiella pneumoniae* and *Staphylococcus aureus* and methanolic extract on *Staphylococcus aureus* and *Streptococcus agalactiae* (Pandya et al., 2019). The metabolites in *P. tuberosa* extracts may be behind the mechanism involved in the antimicrobial action, which may interact with the microbial cell membrane resulting in microbial cell death. The antiulcerogenic activity of aqueous leaf extract of *P. tuberosa* on cold restraint stress, pyloric ligation, and ethanol-induced gastric ulcer rat models was observed. There was significant inhibition in gastric lesions by 76.6% in cold restraint stress, 80.1% in pyloric ligation, and 70.6% in ethanol-induced rat models (Gindi et al., 2010).

In metabolic disorders also, *P. tuberosa* extracts exhibited a hypolipidemic effect. Oral administration of butanol tuber extract of *P. tuberosa* at a dose of 150 mg/kg body weight showed a pronounced protective effect against CCl4-induced hepatotoxicity in adult male rats (Shukla et al., 1996). Rats maintained on high cholesterol diet upon the treatment demonstrated a substantial reduction in serum cholesterol, triglycerides (TG), low-density lipoproteins (LDL), and very-low-density lipoproteins (VLDL) levels (Tanwar et al., 2008). These results were corroborated in another study where nonalcoholic fatty liver disease (NAFLD), induced in rats by feeding a high fat diet, was treated with water extract of this plant. Antioxidant activity with reduced lipid peroxidation and enhanced activities of SOD and catalase enzymes were observed. A similar finding was observed by Tripathi et al. in the NAFLD rats model which also showed a reduction in serum TG and cholesterol values (Tripathi and Aditi, 2020). The ethanolic extract of *P. tuberosa* showed a dose-dependent immunosuppressant activity as evident by a decrease in antibody titer and also a reduction in hematological parameters in the drug-induced myelosuppression model (Babu et al., 2016). Crude powder (3 g daily) of *P. tuberosa* tuber was given to a human patient with ischemic heart disease for twelve months. The case study demonstrated an overall significant cardioprotective effect; resting mean blood pressure was reduced from 96.66 to 90.00 mm Hg without affecting the resting heart rate, and the heart rate at peak exercise was also reduced, indicating better exercise tolerance (Verma et al., 2009).

*P. tuberosa* root extract, given to male Wistar rats (100 mg/rat per day) for 60 days, affected the fertility of rats as shown by a reduction in weight of testes, epididymis, prostate, and the seminal vesicle. Studies also showed a considerable decrease in the quantity of mature Leydig cells, cauda epididymis, and sperm motility (Gupta et al., 2005). The antioxidant-enriched fraction from the tuber extract of *P. tuberosa* against menopausal osteoporosis in ovariectomy-induced osteoporosis in rats was studied and found that it improved biochemical parameters, controlled the increased body weight, and decreased uterus weight following ovariectomy as well as restoration of typical bone structure and trabecular width of the femur (Satpathy et al., 2020). Incision and excision wounds were treated with methanolic and ethyl acetate tuber extract of *P. tuberosa*. The extracts showed potent wound healing property in comparison to the control and the group of rats treated with standard drugs, ibuprofen, and nitrofurazone ointment (Kambhoja and Murthy, 2007).

### Phytochemistry

The crude tuber extracts of *P. tuberosa* are known to contain alkaloids, anthracene, anthocyanidins, anthraquinone, glycosides, carbohydrates, catecholic compounds, coumarins, flavonoids, glycosides, hexose sugars, saponins, steroids, terpenoids, and volatile oils (Ratnam and Venkata Raju, 2009; Rawtal et al., 2019). Therefore, many studies have been undertaken to individually analyze and characterize the activities of different phytoconstituents of the plant. Vaishnav et al. could grow a callus culture of *P. tuberosa* and identified four isoflavanoids, viz., puerarin [1], daidzein [2], genistin [3], and genistein [4] (Vaishnav et al., 2006; Satpathy et al., 2017). Lupinoside PA4 [5] was isolated from methanolic extract of *P. tuberosa* using HPLC, and its structure was determined by 1D, 2D NMR, and Q-TOF-MS (Dey et al., 2007). Pandey and Tripathi extracted tuberosin [6], 3-O-methylanhydrotuberosin [7], and puerarostan [8] from ethanolic tuber extract; the same was confirmed by UV, IR, and NMR spectral data (Pandey and Tripathi, 2010). β-Sitosterol [9] was quantified in the methanolic root extract of *P. tuberosa* by high-performance thin layer chromatography (HPTLC) method (Mhaske et al., 2009). Liquid chromatography–mass spectrometry (LC–MS) analysis of ethanolic extract was found to contain puerarin, daidzein, biochanin A [10], and biochanin B [11] (formononetin) (Chauhan et al., 2013). Daidzin [12], irisolidone [13], 4-methoxypuerarin [14], puerarone [15], quercetin [16], and tectoridin [17] are the flavonoid compounds and p-coumaric acid [18], which have been reported to be isolated from tuber of *P. tuberosa* (Maji et al., 2014) and aqueous tuber decoction shown to contain daidzein, genistin, hydroxytubersone [19], puerarin, puetuberosanol [20], robinin [21], tuberosin, and tuberostan [22] (Shukla et al., 2017). Mass spectrometry and 2D-NMR techniques were used to isolate isoorientin [23] and mangiferin [24] from methanolic extract from *P. tuberosa* (Sumalatha et al., 2015). Phytochemical analysis of *P. tuberosa* extract using HPTLC revealed the presence of carbohydrates, proteins, alkaloids, flavonoids, saponins, phenols, and tannins (Viji and Paulsamy, 2018). Satpathy et al. showed the presence of 23 bioactive molecules including stigmasterol [25], β-sitosterol, and stigmasta-3,5-dien-7-one by gas chromatography–mass spectrometry analysis of antioxidant-enriched fraction prepared from *P. tuberosa* (Satpathy et al., 2020). We have listed various phytoconstituents isolated from *P. tuberosa* and provided detailed information about their chemical structures, IUPAC names, and pharmacological activities, as well as associated references, in Table 2. The chemical structures of phytochemical compounds from *P. tuberosa* were drawn using “ChemDraw JS 19.0”; https://chemdrawdirect.perkinelmer.cloud/js. IUPAC (International Union of Pure and Applied Chemistry) names have been taken from PubChem database.

**TABLE 2:** Pharmacological activities of phytoconstituents of *Pueraria tuberosa*.

Purified compound studied	Model used for study (*in silico/in vitro/in vivo*)	Dose tested	Pharmacological activity	Conclusion	References
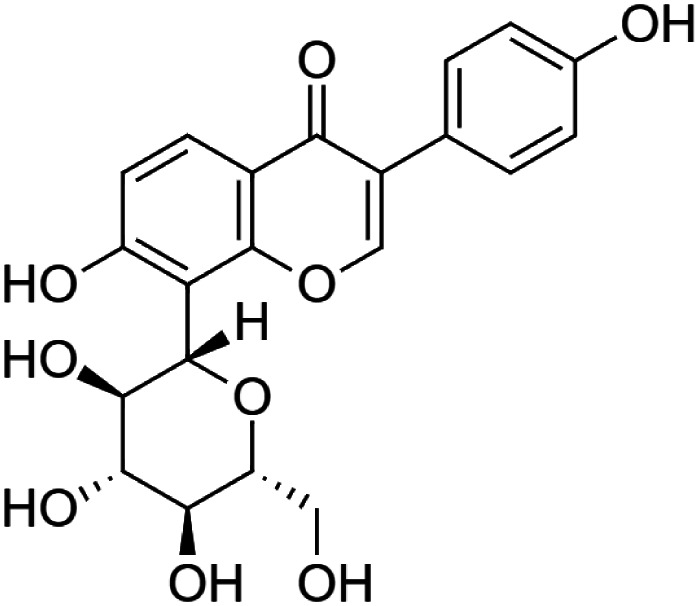 Puerarin **[1]** (C_21_H_20_O_9_) IUPAC name–[7-hydroxy-3-(4-hydroxyphenyl)-8-[(2S,3R,4R,5S,6R)-3,4,5-trihydroxy-6-(hydroxymethyl)oxan-2-yl]chromen-4-one]	*In vivo*	100 mg/kg b/w for 7 days	Nephroprotective	Suppression of oxidative stress production and S-nitrosylation of proteins in the diabetic kidneys and MMP-9	Zhong et al. (2014)
	*In vivo*	20, 40, and 80 mg/kg b/w/day for 8 weeks	Antidiabetic	Hypoglycemic effect which supports its antidiabetic property and renal protective effects via the mechanism of attenuating SIRT1/FOXO1 pathway	Xu et al. (2016)
	*In vivo*	2.5 mg/kg b/w/day for 2 weeks	Antioxidant	Suppressed macrophage activation by inhibiting IκB, ERK, and p38 activity and reactive oxygen species production	Tanaka et al. (2016)
	*In vitro*	10 and 50 µM	Anticancer	Suppressed MCF-7 and MDA-MB-231 cell LPS-stimulated migration, invasion and adhesion by inhibition of the NF-kB pathway and phosphorylation of ERK	Liu et al. (2017)
	*In vivo*	500 mg/kg b/w/day for 6 weeks	Antidiabetic	Improved insulin resistance and reduced diabetic foot ulcers	Yu et al. (2017)
	*In vitro*	0.01, 0.1, 1, 10, and 100 μmol/L	Anticancer	Puerarin-induced apoptosis in human bladder cancer cells was mediated by activation of the mTOR/p70S6K signaling pathway	Jiang et al. (2018)
	*In vivo*	25, 50, and 100 mg/kg b/w/day for 12 weeks	Antidiabetic	Hypoglycemic effects, prevented cataract development and progression in diabetic rats by reducing oxidative stress through the NRF2/HO-1 signaling pathway	Zhang and Li (2019)
	*In vivo*	50 mg/kg b/w/day for 14 weeks	Anti-inflammatory	Reduced inflammatory regulators (TNF-α, IL-1β, COX2, and MMP-14) and inhibited HDAC1/HDAC3 signaling	Guo et al. (2019)
	*In vivo*	5, 10, 20, and 40 mg/kg b/w for 12 weeks	Nephroprotective	Protects podocytes from diabetes-induced injury through HMOX1 and SIRT1-mediated upregulation of autophagy	Li et al. (2020)
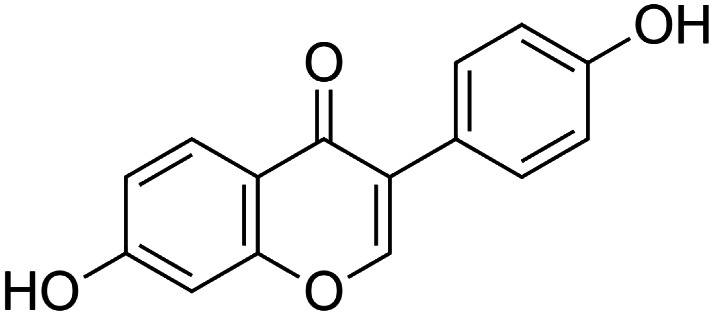 Daidzein **[2]** (C_15_H_10_O_4_) IUPAC name–[7-hydroxy-3-(4-hydroxyphenyl)chromen-4-one]	*In vitro/in vivo*	*In vitro*: 12.50–50 μM; *in vivo*: 1 g/kg b/w for 12 weeks	Anti-inflammatory	Reduced adipose tissue inflammation through the upregulation of PPARγ, which might result in alleviating insulin resistance in obesity	Sakamoto et al. (2014)
	*In vitro/in vivo*	*In vitro*: 0.5–100 µM; *in vivo*: 10 mg/kg and 20 mg/kg b/w for 27 days	Anticancer	Reduced viability of bladder carcinoma RT112 cells by inducing G1/S phase arrest and apoptosis and inhibited tumor growth	He et al. (2016)
	*In vitro/in vivo*	*In vitro*: 12.50–400 μM; *in vivo*: 10 mg/kg and 20 mg/kg b/w for 27 days	Anticancer	Reduced the cell viability and colony formation in concentration-dependent manner and inhibited tumor growth	Zheng et al. (2017)
	*In vitro/in vivo*	*In vitro*: 0.78–200 μM; *in vivo*: 10–40 µg/kg b/w	Anticancer	Induced G2/M cell cycle arrest and suppressed the ovarian tumor growth	Hua et al. (2018)
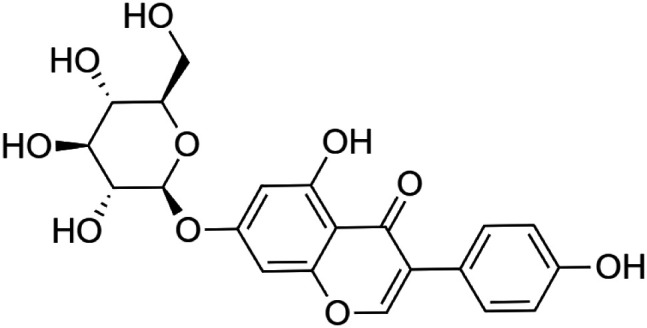 Genistin **[3]** (C_21_H_20_O_10_) IUPAC name–[5-hydroxy-3-(4-hydroxyphenyl)-7-[(2S,3R,4S,5S,6R)-3,4,5-trihydroxy-6-(hydroxymethyl)oxan-2-yl]oxychromen-4-one]	*In vivo*	20, 40, and 60 mg/kg b/w	Cardioprotective	Significantly attenuated the release of LDH, CK in a dose-dependent manner	Gu et al. (2016)
	*In vitro*	50 and 100 µM for 48 h	Antiadipogenic and antilipogenic	Activated AMP-activated protein kinase *a* (AMPKα), and inhibited sterol regulatory element-binding transcription factor-1c (SREBP-1c)	Choi et al. (2020)
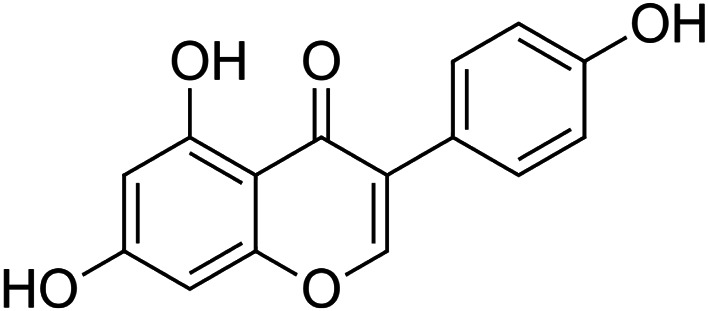 Genistein **[4]** (C_15_H_10_O_5_) IUPAC name–[5,7-dihydroxy-3-(4-hydroxyphenyl)chromen-4-one]	*In vivo*	10 and 20 mg/kg b/w 3 times a week for 10 weeks	Nephroprotective	Reduced renal inflammation, oxidative stress, and apoptosis in diabetic mice	Elmarakby et al. (2011)
	*In vitro*	10 nmol/L to 5 μmol/L	Antidiabetic	Acted on pancreatic β-cells, activation of the cAMP/PKA signaling cascade	Liu et al. (2006)
	*In vivo*	0.2, 1, and 5 mg/kg b/w once daily	Wound healing	Suppression of FoxO1, iNOS activity, and oxidative stress	Tie et al. (2013)
	*In vitro*	5, 10, and 25 µM for 24 h	Antioxidant	Activated AMPK and increased PTEN expression	Park et al. (2010)
	*In vivo*	2.5–10 mg/kg b/w for 14 days	Neuroprotective	Reduced the infarct volume, improved the neurological deficit, and prevented cell apoptosis after ischemia	Qian et al. (2012)
	*In vivo*	10 mg/kg b/w 1 h before surgery	Nootropic	Ameliorated aβ-induced impairment of short-term spatial memory in rats through an estrogenic pathway and reduced oxidative stress	Bagheri et al. (2011)
	*In vivo*	1 mg/kg b/w from day 16 until day 60	Anti-stress	Lowered blood pressure, restored ACE, PKC-bII, and eNOS expression, and preserved renal ultrastructural integrity	Palanisamy and Venkataraman (2013)
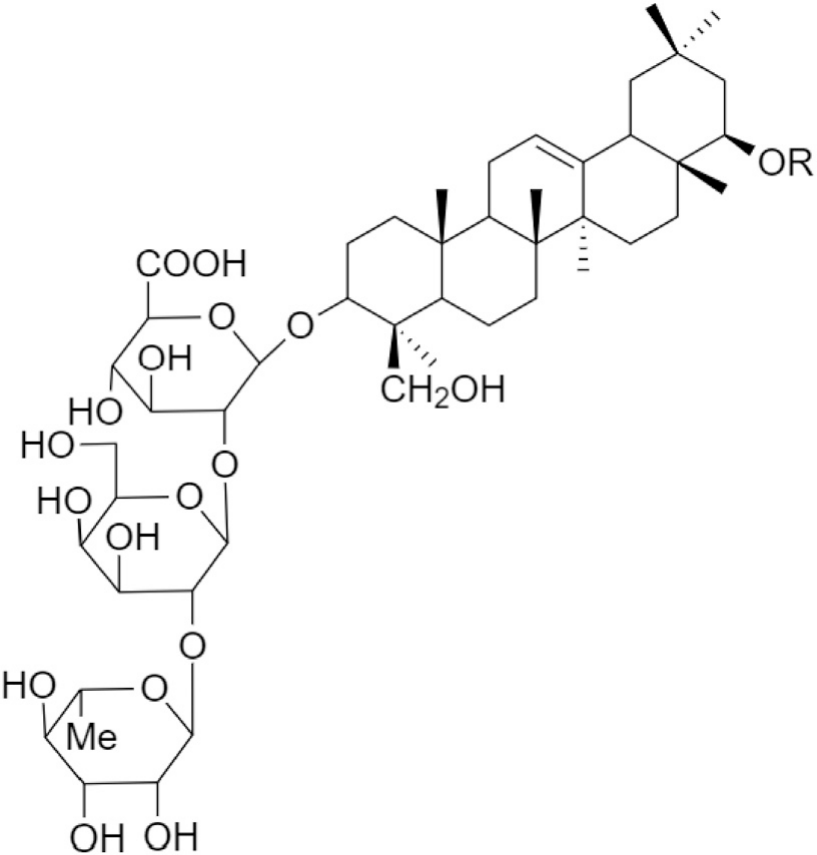 Lupinoside PA4 **[5]** Dey et al. (2007)	*In vitro/in vivo*	*In vitro*: 20 ng/ml; *in vivo*: 1.5 mg/200 g b/w for 12 days	Antidiabetic	Stimulated IR-β and akt phosphorylation	Dey et al. (2007)
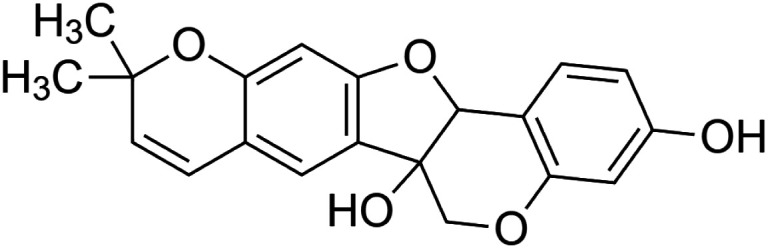 Tuberosin **[6]** (C_20_H_18_O_5_) IUPAC name–[7,7-dimethyl-8,12,20-trioxapentacyclo [11.8.0.0^2,11^.0^4,9^.0^14,19^]henicosa-2 (11),3,5,9,14 (19),15,17-heptaene-1,17-diol]	*In vitro*	50, 100, 300, and 600 ng/ml	Antioxidant	Inhibited LPS-induced NO production in a concentration-dependent manner, expression of iNOS proteins	Pandey and Tripathi (2010)
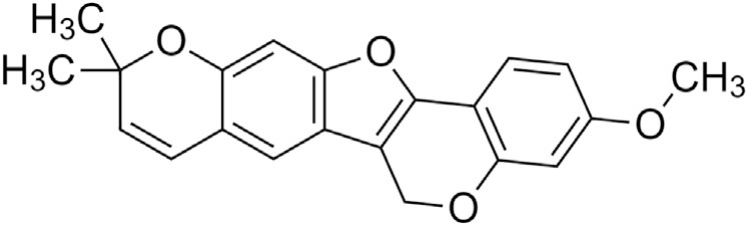 3-O-methylanhydrotuberosin **[7]** (C_21_H_18_O_4_) IUPAC name–[17-methoxy-7,7-dimethyl-8,12,20-trioxapentacyclo [11.8.0.0^2,11^.0^4,9^.0^14,19^]henicosa-1(13),2(11),3,5,9,14 (19),15,17-octaene]	Pharmacological activity not reported				
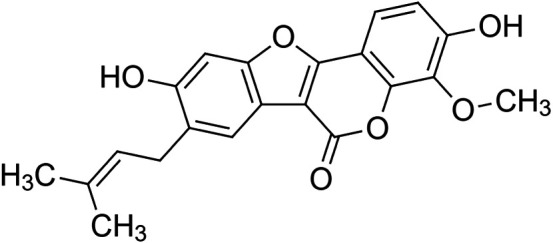 Puerarostan **[8]** (C_21_H_18_O_6_) IUPAC name–[3,9-dihydroxy-4-methoxy-8-(3-methylbut-2-enyl)-[1]benzofuro [3,2-c]chromen-6-one]	Pharmacological activity not reported				
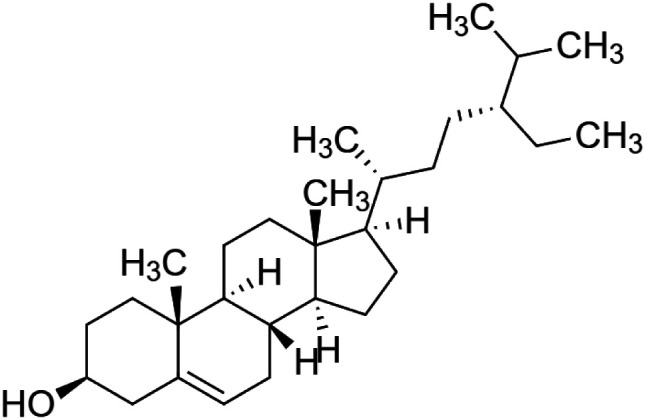 β-sitosterol **[9]** (C_29_H_50_O) IUPAC name–[(3S,8S,9S,10R,13R,14S,17R)-17-[(2R,5R)-5-ethyl-6-methylheptan-2-yl]-10,13-dimethyl-2,3,4,7,8,9,11,12,14,15,16,17-dodecahydro-1H-cyclopenta [a]phenanthren-3-ol]	*In vivo*	20 mg/kg b/w	Anti-colitis	Ameliorated HFD-induced colitis by inhibiting the binding of LPS to toll-like receptor 4 in the NF-κB pathway	Kim et al. (2014)
	*In vitro*	0.25 µg/ml and 2.5 µg/ml	Antiproliferative for mast cell	Decreased thymic stromal lymphopoietin (TSLP) induced mast cell proliferation	Han et al. (2015)
	*In vivo*	20 mg/kg b/w	Nephroprotective	β-sitosterol showed significant positive changes to nephrotoxicity-induced rats; altered biochemical parameters were restored to near normal	Sharmila et al. (2016)
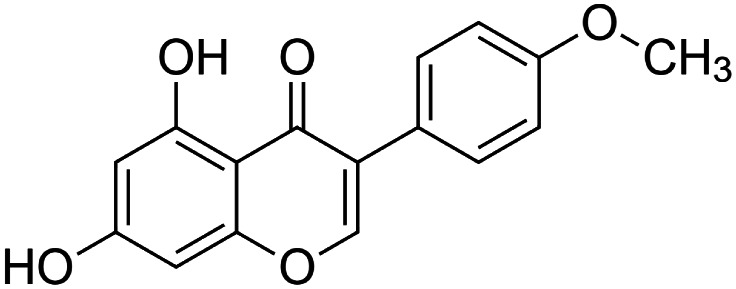 Biochanin a **[10]** (C_16_H_12_O_5_) IUPAC name–[5,7-dihydroxy-3-(4-methoxyphenyl)chromen-4-one]	*In vivo*	10, 20, and 40 mg/kg b/w for 28 days	Antidiabetic	Increased NAD-dependent deacetylase sirtuin-1 (SIRT1) expression in pancreatic tissue	Oza and Kulkarni (2018b)
	*In vitro*	5–20 μM for 1 h	Antitoxic against 2,3,7,8-tetrachlorodibenzo-p-dioxin	Inhibited the TCDD-induced loss of triglycerides in 3T3-L1 adipocytes, showing increased differentiation of 3T3-L1 preadipocytes to adipocytes when compared with the cells exposed to TCDD alone	Choi et al. (2019)
	*In vitro*	2–4 µM	Vasodilatory	Interfered with the cGMP pathway in isolated coronary arteries and vasodilatory effect	Migkos et al. (2020)
	*In vitro*	2.5–100 μM	Anti-inflammatory	Suppressing iNOS, COX-2, MyD88, and TLR-4 protein expressions and akt and ERK1/2 pathway activation	Berköz et al. (2020)
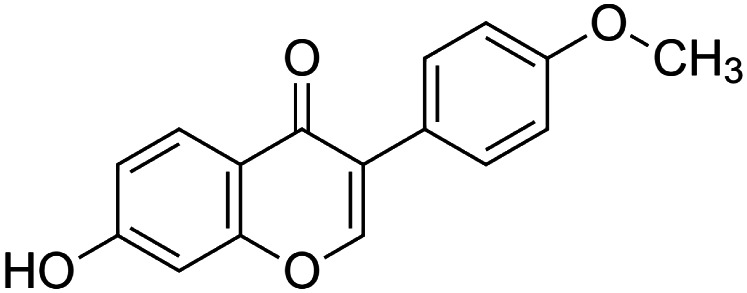 Biochanin B **[11]** (formononetin) (C_16_H_12_O_4_) IUPAC name–[7-hydroxy-3-(4-methoxyphenyl)chromen-4-one]	*In vitro*	3.45 μmol/L in Vero cells and 3.98 μmol/L in SK-N-SH cells	Anti-enterovirus 71	Inhibited EV71-induced COX-2 expression and PGE2 production via MAPKs pathway including ERK, p38, and JNK	Wang et al. (2015)
	*In vivo*	1 and 100 µM for 2 weeks	Hair growth activity	Topical formononetin treatment induced hair regrowth in the depilated telogenic C57BL/6 mice and restored the length of hair shafts and size of hair follicles	Kim et al. (2016)
	*In vivo*	5, 10, and 20 mg/kg b/w	Antidiabetic	Inhibited apoptosis of β-cell of the pancreas and promoted islet β-cell regeneration	Qiu et al. (2017)
	*In vivo*	15, 50, and 75 mg/kg b/w for 5 days	Nephroprotective	Promoted proliferation of surviving renal tubular cells and inhibited apoptosis after cisplatin-induced acute kidney injury	Huang et al. (2017)
	*In vitro*	0–280 μM for 24 and 48 h	Anticancer	Significant increase in Bax/Bcl-2 ratio accompanied with elevated level of cleaved-caspase-3 and cleaved-caspase-9 after formononetin treatment	Zhang et al. (2018)
	*In vitro*	2.5, 5, and 10 µM for 24 h	Neuroprotective	Inhibited neuroinflammation in BV2 microglia cells stimulated with LPS and also suppressed production of the proinflammatory cytokines TNF-α, IL-6, and IL-1β from the cells	El-Bakoush and Olajide (2018)
	*In vivo*	10, 20, and 40 mg/kg b/w for 28 days	Antidiabetic	Reduced insulin resistance and attenuated hyperglycemia in type II diabetes, which could be due to increased expression of SIRT1 in pancreatic tissues	Oza and Kulkarni (2018a)
	*In vitro*	30 μM for 24 h	Anti-inflammatory	Inhibited HMGB1 release by decreased HMGB1 acetylation via upregulating SIRT1 in a PPARδ-dependent manner	Hwang et al. (2018)
	*In vitro*	5 mM	Cardioprotective	Pretreatment with formononetin reduced myocardial tissue injury, improved cardiac function, and decreased apoptosis in heart tissue	Huang et al. (2018)
	*In vitro*	25–100 µM for 24 h	Nephroprotective	Formononetin-treated cells were morphologically normal compared to the cells undergoing cisplatin-induced death and showed potent protective effect against cisplatin-mediated LLC-PK1 cell (renal tubular epithelial cell) death	Lee et al. (2018)
	*In vivo*	25, 50, and 100 mg/kg b/w for 8 days	Anti-colitis	After formononetin administration, there was less infiltration of neutrophils and macrophages in the injured colonic tissue and also a significant decrease in the level of inflammatory cytokines TNF-α and IL-1β in the colon of mice with acute colitis	Wu et al. (2018)
	*In vitro/in vivo*	*In vitro*: 150 μmol/L for 12, 24, and 48 h; *in vivo*: 50 mg/kg b/w for 4 weeks	Anticancer	Inhibited MDA-MB-468 cell survival in a dose- and time-dependent manner, and tumor volume shrank from 472.7 to 253.6 mm^3^ on day 30 in xenograft model	Zhou et al. (2019)
	*In vivo*	10 mg/kg b/w	Anticancer	The tumor inhibition rate was 50.17% in the mice treated with formononetin	Zhang et al. (2019)
	*In vivo*	100 mg/kg b/w for 14 weeks	Hepatoprotective	Promoted lysosome biogenesis and autophagosome-lysosome fusion, relieving the blockade in autophagic flux and further induced lipophagy	Wang et al. (2019)
	*In vivo*	10–50 mg/kg b/w for 10 days	Hepatoprotective	Ameliorated hepatic cholestasis by upregulating expression of SIRT1 and activating PPARa	Yang et al. (2019)
	*In vivo*	20 and 40 mg/kg b/w for 10 weeks	Neuroprotective	Reduced the levels of inflammation cytokines IL-1β and TNF-α and tau hyperphosphorylation in mice hippocampus	Fu et al. (2019)
	*In vivo*	31.25 μg/ml	Anti-inflammatory	LPS-induced inflammation in zebrafish was attenuated by formononetin mainly by restraining the MyD88 or TRIF MAPK/ERK and MAPK/JNK pathways	Luo et al. (2019)
	*In vivo*	25 mg/kg b/w for 10 days	Anti-stress	Reduced the neural excitability and the protective upregulation of GABA_A_ receptors	Wang et al. (2019)
	*In vivo*	10, 20, and 40 mg/kg b/w for 16 weeks	Nephroprotective	Enhanced creatinine clearance and reduced oxidative stress burden along with increased SIRT1 expression in kidney tissues	Oza and Kulkarni (2019)
	*In vivo*	10 mg/kg b/w	Anticancer	Inhibited EGFR-Akt axis and promoted FBW7-mediated Mcl-1 ubiquitination	Yu et al. (2020)
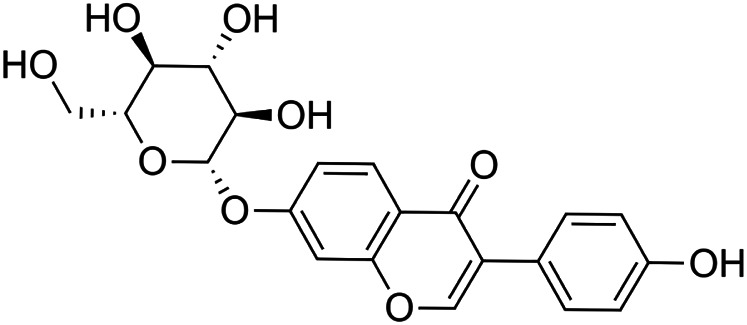 Daidzin [**12]** (C_21_H_20_O_9_) Daidzin, daidzein and their metabolites, O-desmethylangolensin (O-DMA) and equol IUPAC name (Daidzin)–[3-(4-hydroxyphenyl)-7-[(2S,3R,4S,5S,6R)-3,4,5-trihydroxy-6-(hydroxymethyl)oxan-2-yl]oxychromen-4-one]	*In vitro*	5–100 µM	Antioxidant	Stimulated catalase and total superoxide dismutase (CuZn- and Mn-SOD) activity, and mRNA and protein expression	Choi and Kim (2014)
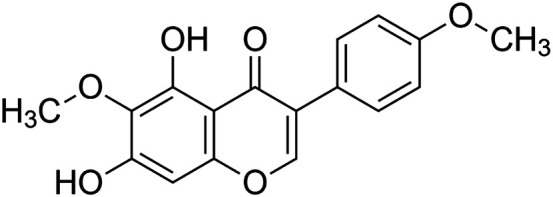 Irisolidone **[13]** (C_17_H_14_O_6_) IUPAC name–[5,7-dihydroxy-6-methoxy-3-(4-methoxyphenyl)chromen-4-one]	*In vitro/in vivo*	*In vitro*: 1, 5, and 10 μM for 12 h; *in vivo*: 50, 100, and 200 mg/kg b/w for 30 days	Anti-ischemia	*In vitro*, increased cell viability and attenuated apoptosis; *in vivo*, inhibited mitochondrial membrane potential (MMP) and increased total ATPase activity	Yin et al. (2016)
	*In vitro/in vivo*	*In vitro*: 5 or 10 μM for 90 min; *in vivo*: 20–50 mg/kg for 4 days	Anti-gastritic	Pretreatment with irisolidone decreased the area of hemorrhagic ulcerative lesions caused by ethanol and suppressed stomach myeloperoxidase activity, CXCL4 secretion, and NF-κB activation	Kang et al. (2017)
	*In vivo*	20 mg/kg b/w	Anti-colitic	Alleviated colon shortening and myeloperoxidase activity in mice with TNBS-induced colitis	Jang et al. (2019)
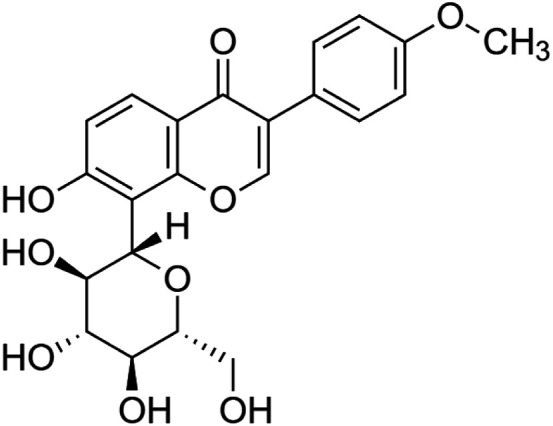 4-Methoxypuerarin **[14]** (C_22_H_22_O_9_) IUPAC name–[7-hydroxy-3-(4-methoxyphenyl)-8-[(2S,3R,4R,5S,6R)-3,4,5-trihydroxy-6-(hydroxymethyl)oxan-2-yl]chromen-4-one]	*In silico*	—	Weak DNA binding affinity	Glycosylation of 4′-methoxypuerarin, caused steric hindrance to weaken the DNA binding affinity and had no significant inhibition on DNA amplification	Chen et al. (2020)
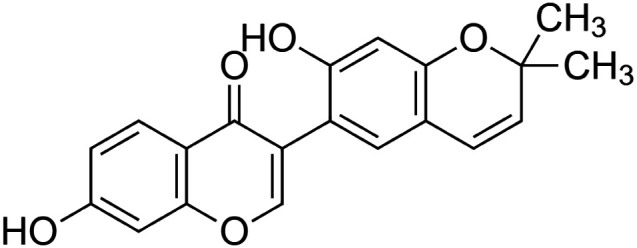 Puerarone **[15]** (C_20_H_16_O_5_) IUPAC name–[7-hydroxy-3-(7-hydroxy-2,2-dimethylchromen-6-yl)chromen-4-one]	*In silico*	—	Antidiabetic	Strong affinity to VEGFR-1 and VEGFR-2 along with 93.881% human intestinal absorption	Srivastava et al. (2017)
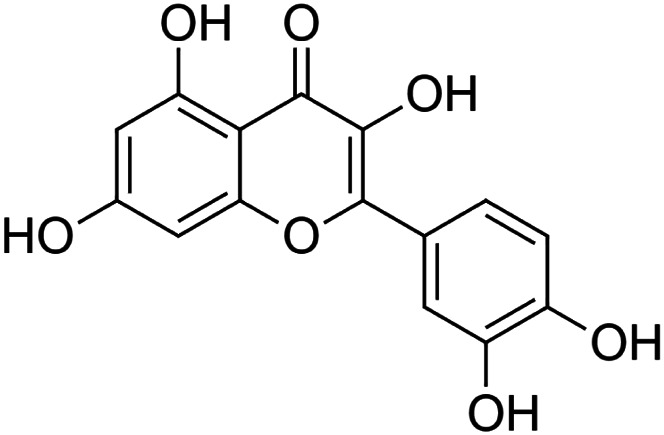 Quercetin **[16]** (C_15_H_10_O_7_) IUPAC name–[2-(3,4-dihydroxyphenyl)-3,5,7-trihydroxychromen-4-one]	*In vivo*	15 mg/kg b/w for 7 days	Hepatoprotective	Accelerated the regeneration after partial hepatectomy	Kanter et al. (2016)
	*In vitro/in vivo*	*In vitro:* 5–100 μM; *in vivo*: 100 mg/kg b/w for 30 days	Neuroprotective	Protected neuronal cells from amyloid beta induced oxidative stress	Li et al. (2017)
	*In vivo*	100 mg/kg b/w for 6 days	Intestinal damage repair	Increased intestinal and mucosal weight and prevented methotrexate-induced intestinal damage	Sukhotnik et al. (2018)
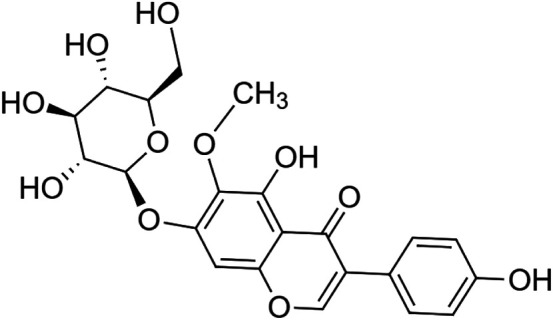 Tectoridin **[17]** (C_22_H_22_O_11_) IUPAC name–[5-hydroxy-3-(4-hydroxyphenyl)-6-methoxy-7-[(2S,3R,4S,5S,6R)-3,4,5-trihydroxy-6-(hydroxymethyl)oxan-2-yl]oxychromen-4-one]	*In vivo*	25–400 mg/kg b/w	Anti-alcoholism	Strongest clearance rate of ethanol	Zhang et al. (2019)
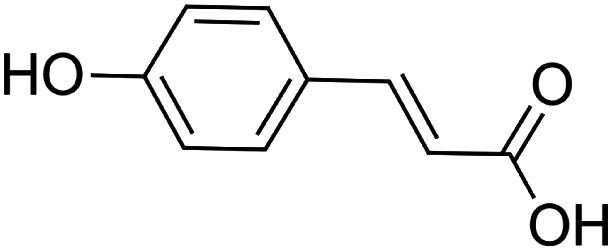 *p*-coumaric acid **[18]** (C_9_H_8_O_3_) IUPAC name–[(E)-3-(4-hydroxyphenyl)prop-2-enoic acid]	*In vivo*	100 mg/kg b/w for 7 days	Immunomodulatory	Decreased the expression of inflammatory mediator TNF-α and circulating immune complexes	Pragasam et al. (2013)
	*In vivo*	8 mg/kg b/w for 7 days	Cardioprotective	Prevented cardiac hypertrophy, by virtue of its antihypertrophic, antilipidemic, and free radical scavenging	Roy and Prince (2013)
	*In vivo*	100 mg/kg b/w for 3 weeks	Nephroprotective	Cadmium metal chelating activity	Navaneethan and Rasool (2014)
	*In vivo*	100 mg/kg b/w	Antidiabetic	Modulated glucose and lipid metabolism via GLUT2 activation in the pancreas	Amalan et al. (2016)
	*In vivo*	30 mg/kg b/w	Neuroprotective	Increased the total activity of fEPSP dose-dependently after high frequency stimulation and attenuated scopolamine-induced blockade of fEPSP in the hippocampal CA1 long-term potentiation area	Kim et al. (2017)
	*In vivo*	100 mg/kg b/w for 26 days	Anti-arthritic	Suppressed the paw edema, body weight loss and inflammatory cytokine and chemokine levels (TNF-α, IL-1β, IL-6, and MCP-1) in serum and ankle joint of arthritic rats	Neog et al. (2017)
	*In vivo*	50 mg/kg b/w	Hepatoprotective	Suppressed hepatic apoptosis via ROS-mediated DNA damage and inflammation by modulating the mitogen-activated protein kinase (MAPK) signaling axis in an ROS-dependent manner	Cha et al. (2018)
	*In vivo*	100 mg/kg b/w for 2 weeks	Neuroprotective	Pretreatment with p-coumaric acid significantly reduced malondialdehyde (MDA) levels, whole-brain infarction volume, and hippocampal neuronal death together and increased catalase and superoxide dismutase activities	Sakamula and Thong-asa (2018)
	*In vitro/in vivo*	*In vitro*: 0–4,000 µmol/L for 24 and 72 h; *in vivo*: 100 mg/kg b/w for 30 weeks	Anticancer	Downregulated Grp78 and activated UPR mediated apoptosis both in *vitro* and *in vivo* models of colon cancer	Sharma et al. (2018)
	*In vivo*	50 and 100 µmol/L	Antioxidant	Significantly increased the survival rate of *Caenorhabditis elegans* under the oxidative stress condition and also increased lifespan by 20% for both 50 and 100 µmol/L compared to the control	Yue et al. (2019)
	*In vivo*	50 mg/kg b/w for 6 weeks	Antidiabetic	Enhanced anti-inflammatory, anti-osteoclastogenic, and antioxidant defense systems in streptozotocin-treated mice	Bhattarai et al. (2019)
	*In vivo*	*In vitro*: 10–100 μM; *in vivo*: 50, 100, and 200 mg/kg b/w	Hepatoprotective	No effect on cell viability up to 60–80 μM concentrations on HepG2 cells *in vitro*, p-coumaric acid at 200 mg/kg exhibited higher protection on ethanol-induced hepatic injury in rats	Sabitha et al. (2020)
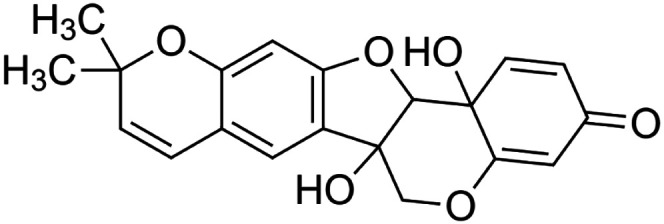 Hydroxytuberosone **[19]** (C_20_H_18_O_6_) IUPAC name–[1,14-dihydroxy-7,7-dimethyl-8,12,20-trioxapentacyclo [11.8.0.0^2,11^.0^4,9^.0^14,19^]henicosa-2 (11),3,5,9,15,18-hexaen-17-one]	*In vivo*	Topical application	Wound healing	Excision and incision wound model	Kambhoja and Murthy (2007)
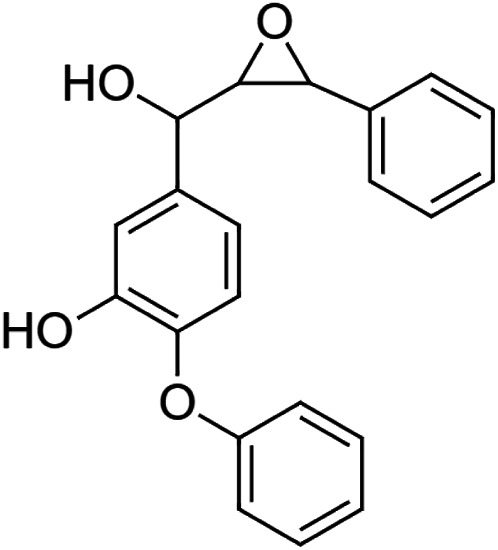 Puetuberosanol **[20]** (C_21_H_18_O_4_) IUPAC name–[5-[hydroxy-(3-phenyloxiran-2-yl)methyl]-2-phenoxyphenol]	Pharmacological activity not reported				
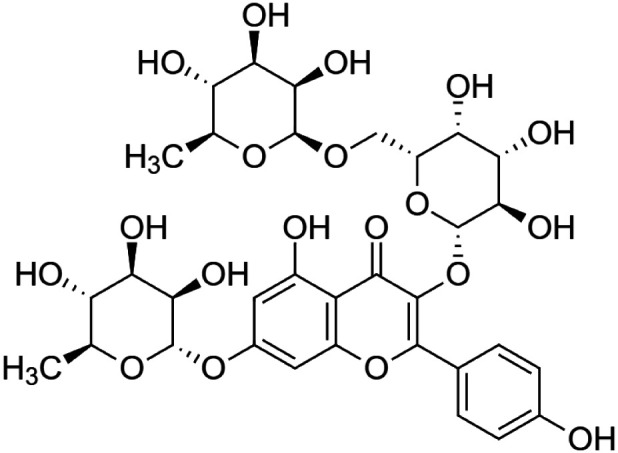 Robinin **[21]** (C_33_H_40_O_19_) IUPAC name–[5-hydroxy-2-(4-hydroxyphenyl)-7-[(2S,3R,4R,5R,6S)-3,4,5-trihydroxy-6-methyloxan-2-yl]oxy-3-[(2S,3R,4S,5R,6R)-3,4,5-trihydroxy-6-[[(2S,3R,4R,5R,6S)-3,4,5-trihydroxy-6-methyloxan-2-yl]oxymethyl]oxan-2-yl]oxychromen-4-one]	*In vivo*	50 mg/kg b/w for 10 days	Cardioprotective	Modulation of TGF-β1 signaling pathway in doxorubicin-induced cardiac toxicity in Sprague Dawley rats	Janeesh and Abraham (2014)
	*In vitro*	6 μg/ml	Immunomodulatory	Inhibited TLR4-NF-κB signaling pathway	Janeesh et al. (2014)
	*In vitro*	0.125–0.50 mg/ml	Antioxidant	The total antioxidant capacity (TAC) in robinin was significantly higher and best maintained the follicular morphology	Dos Santos Morais et al. (2019)
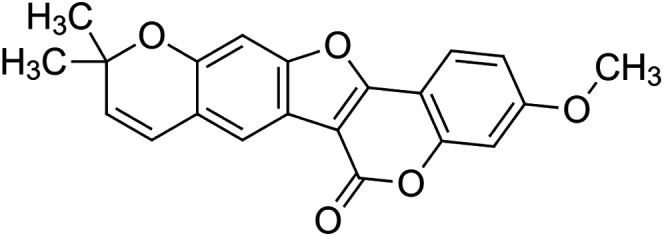 Tuberostan **[22]** (C_21_H_16_O_5_) IUPAC name–[17-methoxy-7,7-dimethyl-8,12,20-trioxapentacyclo [11.8.0.0^2,11^.0^4,9^.0^14,19^]henicosa-1(13),2(11),3,5,9,14 (19),15,17-octaen-21-one]	*In silico*	—	Antidiabetic	In molecular docking study, tuberostan showed best interaction for GLP-1R with binding energy at 8.15 kcal/mol and dissociation constant at 1061624.125 pM	Srivastava et al. (2018)
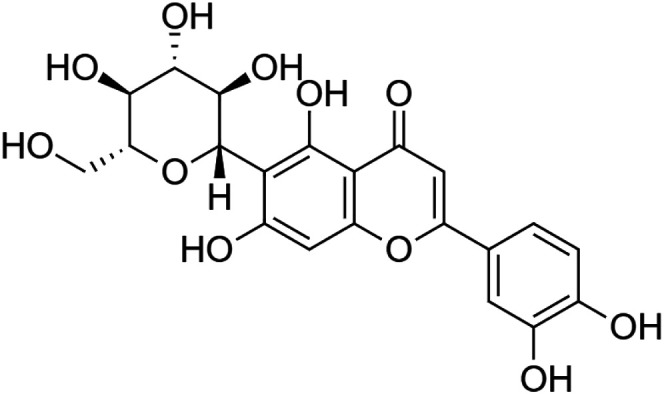 Isoorientin **[23]** (C_21_H_20_O_11_) IUPAC name–[2-(3,4-dihydroxyphenyl)-5,7-dihydroxy-6-[(2S,3R,4R,5S,6R)-3,4,5-trihydroxy-6-(hydroxymethyl)oxan-2-yl]chromen-4-one]	*In vitro*	0.1–100 µM	Anti-inflammatory	Inhibited COX-2 activity by 64%	Sumalatha et al. (2015)
	*In vitro/in vivo*	*In vitro*: 25 nM and 100 μM for 16 hours; *in vivo*: 10 mg/kg and 20 mg/kg b/w for 24 h	Anti-inflammatory	Inhibited the expression of COX-2 *in vitro* and decreased the expression of COX-2, TNF-α, IL-1β, iNOS, and 5-LOX in dose-dependent manner in carrageenan-induced inflammation in mice	Anilkumar et al. (2017)
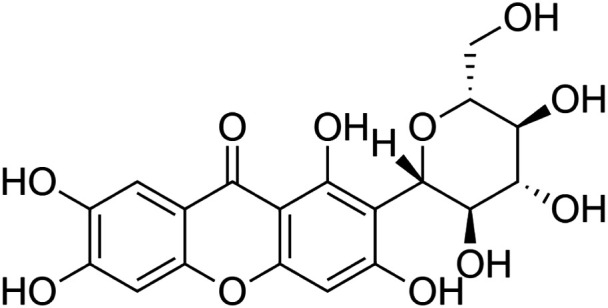 Mangiferin **[24]** (C_19_H_18_O_11_) IUPAC name–[1,3,6,7-tetrahydroxy-2-[(2S,3R,4R,5S,6R)-3,4,5-trihydroxy-6-(hydroxymethyl)oxan-2-yl]xanthen-9-one]	*In vitro*	100 µM	Anti-inflammatory	Inhibited COX-1 and COX-2 activity by 79.4% and 45.9%, respectively	Sumalatha et al. (2015)
	*In vitro/in vivo*	*In vitro*: 20 and 40 μM for 16 h; *in vivo*: 10 mg/kg and 20 mg/kg b/w for 24 h	Anti-inflammatory	Reduced expression of inflammatory mediator (COX-2, iNOS, and TNF-α) and increased anti-inflammatory cytokine (IL-10) and increased size of blood vessels were significantly reduced, and cell infiltration was less compared to mice treated with carrageenan alone	Bulugonda et al. (2017)
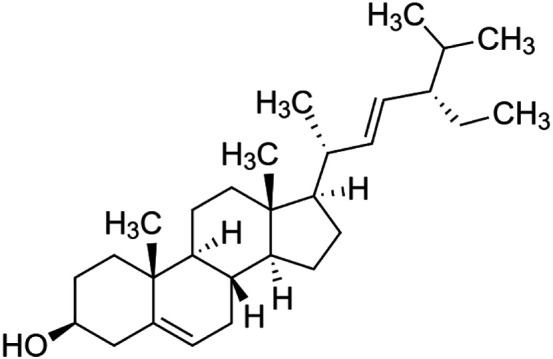 Stigmasterol **[25]** (C_29_H_48_O) IUPAC name–[(3S,8S,9S,10R,13R,14S,17R)-17-[(E,2R,5S)-5-ethyl-6-methylhept-3-en-2-yl]-10,13-dimethyl-2,3,4,7,8,9,11,12,14,15,16,17-dodecahydro-1H-cyclopenta [a]phenanthren-3-ol]	*In vivo*	200 mg/kg and 400 mg/kg b/w	Chemo-preventive	Induced a significant decrease in 7,12-dimethylbenz[a]anthracene (DMBA)-induced skin tumor	Ali et al. (2015)

b/w: body weight.

### Toxicology of Pueraria tuberosa

The acute (single dose of 2,000 and 5,000 mg/kg body weight) and repeated dose (250, 500, 1,000, and 2,000 mg/kg body weight for 28 days) toxicity studies with water extract of the tuber of *P. tuberosa* were conducted in rats as per OECD (Organization for Economic Co-Operation and Development) guidelines. The survival rate and biochemical and histological changes were studied. No adverse effect was reported in single-dose acute toxicity, but in repeated dose toxicity studies, 100% mortality was observed on day 21 at 2,000 mg/kg body weight, and histological examination of the visceral organs showed that this mortality could be due to hepatotoxicity (Pandey et al., 2018). However, histological evaluation of different organs using hematoxylin and eosin staining did not observe any morphological alterations in the spleen, adrenal glands, and heart. The size and shapes in crypts and villi of the intestine and semeniferous tubules were intact with normal spermatozoa count in testis (Pandey et al., 2019). In another experiment on acute toxicity study of poly-herbal formulation (containing *P. tubrosa*), “Dhatryadi Ghrita” methanolic extract did not show any untoward effects in mice (Pal and Mishra, 2019).

## Conclusion and Future Directions

The scientific community worldwide has shown an interest in discovering the disease combating potential of natural flora and bioactive compounds therein. A wide pool of literature suggests that these phytochemicals hold the immense potential of eliminating diseases, and many such plant-based drugs have long been used in many parts of the world. Markedly, the tuber and leaf of *P. tuberosa* plant have been used from ancient times in the traditional practices. Previous literature has shown that leaf and tuber extracts of the plant contain several bioactive constituents that possess an extensive range of pharmacological activities. Some of the isolated compounds, namely, puerarin, irisolidone, genistein, daidzein, biochanin A, biochanin B, isoorientin, and mangiferin, have been studied for various medicinal purposes and demonstrated several pharmacological activities like anticancerous, antidiabetic, anti-inflammatory, antioxidant, antiviral, cardioprotective, fibrinolytic, hepatoprotective, hypolipidemic, immunomodulatory, neuroprotective, nephroprotective, nootropic, vasodilatory, and wound healing. The bioactive constituents of *P. tuberosa* can individually or synergistically exert their therapeutic effects. Apart from puerarin, daidzein, genistein, irisolidone, and biochanin, many more compounds have been identified from *P. tuberosa*; however, underlying mechanisms of action of compounds isolated from this plant are not completely known. Thus, exploration of pharmacological mechanisms of individual bioactive constituents and their toxicity/clinical studies shall be the focus of future investigations. The extensive range of pharmacological properties of *P. tuberosa* could provide us a new interesting path for future research and may present new perspectives for the disease management.
